# China’s decarbonization requires achievable deep underground research facilities

**DOI:** 10.1186/s40562-023-00265-y

**Published:** 2023-02-27

**Authors:** Zhaoxiang Chu, Yiming Wang

**Affiliations:** 1 School of Mechanics and Civil Engineering, China University of Mining and Technology, Xuzhou, China; 2 State Key Laboratory for Geo-Mechanics and Deep Underground Engineering, China University of Mining and Technology, Xuzhou, China

**Keywords:** Geoscience, Energy transition, Climate responsibility, Challenges, DUSEL

## Abstract

This letter introduces the founding of a new Deep Underground Science and Engineering Laboratory-DUSEL in China, the distinguishing feature of which is focusing on the key scientific issue on the law of fluid matter migration within the Earth’s Critical Zone in geoscience. Various technical, economic, and social challenges were elucidated. The achievements of this facility and thereby ambitious research may provide essential solutions to both energy transition and climate security, and then bolster support for decarbonization of China’s energy sector, finally helping attain its double carbon’ goal.

**Graphical Abstract:**

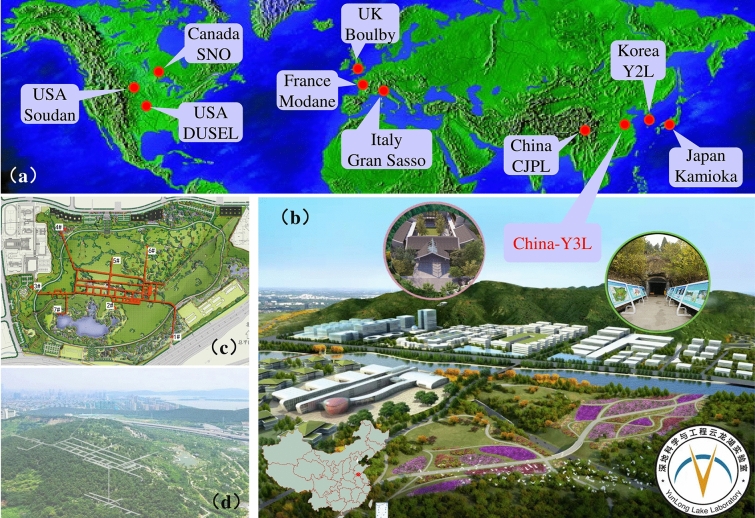

The past 2022 made the carbon energy-related climate issue a global hotspot again due to the military action by Russia in Ukraine (Thorp 2022). This war constrainedly opens a way for fossil fuel-dependent countries to deeply understand the importance of breaking free from carbon energy and accelerating energy transition from carbon intensive to clean alternatives. People should aware that geoscience-oriented energy decarbonization is one essential solution to both energy and climate security.

China is the world’s largest energy consumer [5.24 billion tonnes of standard coal in 2021 (SSB 2022)] and CO_2_ emitter [11.9 billion tonnes in 2021(IEA 2022)], accounting for more than a quarter of global total CO_2_ emissions. Therefore, China’s decarbonization, especially the energy sector, significantly contributes to realizing the Paris climate target. Fortunately, China has pledged to peak its CO_2_ emissions by 2030 and achieve carbon neutrality by 2060 (the so-called double carbon’ goal (Shi et al. 2021)), bearing the responsibility of addressing climate change as a great power. To do this, many substantive actions, both large- and small-scale research projects (O’Meara and Ye 2022), nationwide and world’s largest carbon-trading scheme (Liao and Yao 2022), as well as green belt and clean road initiative (Zhang et al. 2017), have been taken to make China a global leader in climate responsibility. In addition, a new DUSEL, similar with those implemented in Japan, Italy, Canada, and the USA (Fig. 1a), has been instigated in China, aiming to bolster support for decarbonization of the energy sector and help attain its double carbon’ goal. However, such a DUSEL can only achieve success after sufficiently considering various technical, economic, and social challenges.

**Fig. 1:**
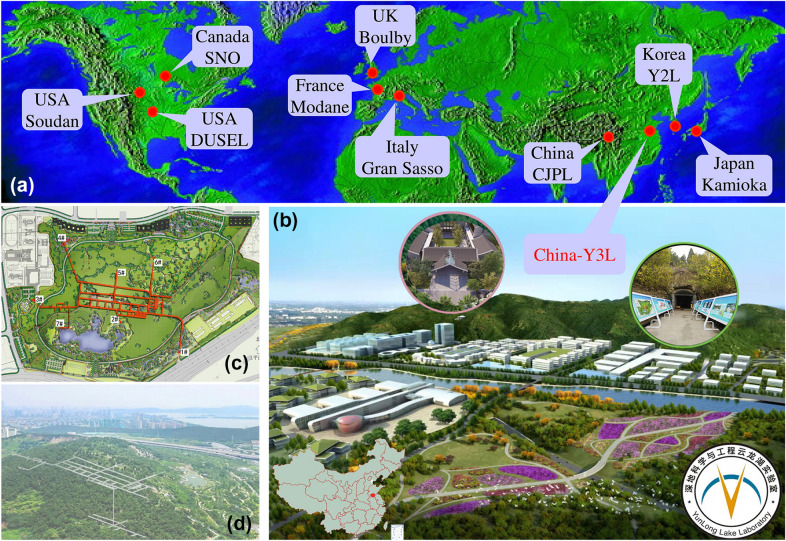
Typical deep underground labs all over the world (**a**) as well as the artist’s rendition and layout of the Y3L (**b**–**d**) near Xuzhou’s scenic YunLong Lake

The YunLong Lake Laboratory (Y3L), the official name of this new DUSEL, mainly contains three parts (Fig. 1b–d): mountain massif tunnel, deep abandoned subsurface mine and ground industrial incubator base. Different from traditional DUSELs with excessive multidisciplinary characters [Physics, Biology, Geoscience and Engineering etc. (Dalton 2009)], we urge the Y3L to focus more on geoscience. The reason is that we are witnessing rapid expansion of energy-related geoscientific technologies—such as earth contact energy storage (compressed air and hydrogen), CO_2_/nuclear waste geological sequestration and geothermal exploitation—which are pivotal and have been treated as promising solutions to decarbonization (Stephenson et al. 2019) and sustainability (Scown 2020). Taking geothermy as an example (Chu et al. 2021), a full life cycle mine-oriented geothermal system within the Earth’s Critical Zone could support energy decarbonization of the Y3L due to the fact that energy demand during the construction, operation and maintenance of a DUSEL itself is expected to be met with renewable sources. All these technologies face a general challenge: the key scientific issue on the law of Earth’s fluid matter migration. Thus, open scientific and technical researches are necessary. They should and can support energy decarbonization not only for the Y3L itself, but also at corporate, industry, even the whole country levels (Aldrich et al. 2019; Wade & Rekker 2020).

Ambitious plans generally require substantial funds. Previous DUSEL built in the USA needs US$700–800 million in total (Reich 2010), while an overall cost of RMB 6 billion is committed to develop the Y3L during China’s Five-Year Plan for 2021–2025. For this financial obstacle, we hold opinions that these pledges ought to be meaningful and a multipartite joint support is preferable. First, the absence of high-energy physics study within the Y3L can save a large part of costs, which in turn greatly improve the likelihood of promised investment. Second, the organizational pattern of the Y3L-government predominant, enterprises and universities involved—can effectively avoid the frustration of denied funding (Reich 2010); thus, guaranteeing its economic viability.

Scientific community usually pays insufficient attention to the social threat, which, however, is the overriding element resulting in pause, delay and failure, even expedited success of a DUSEL construction. First, cooperation with the local residents is vital to keeping the Y3L on track (Dalton 2009). Any harm yielded from the Y3L to personnel and environment (noise, radiation and vibration) is not allowed, but meanwhile the Y3L is always required to be able to provide intensive outreach service such as additional job opportunity for local tribes. If not, this type of project could face trouble. Second, changes in the social policy can also have positive effects. The Y3L is a response of the reconstruction plan of China's state laboratory system, the main purpose of which is enhancing China’s ability to innovate in science and technology for energy, economy and climate, as well as respond to emergencies and changes on the institutional level (e.g. COVID-19). Such a top-down overhaul will affect investment delivered to this field and thereby the Y3L. However, these policy-induced transformation must be inclusive, just like Poland’s gradual energy transition after it was accepted by the EU (Woniak and Pactwa 2019), and can be accelerated, but not radical (Shi et al. 2021), let along geopolitical will deduced wars (Thorp 2022).

Totally, a new DUSEL in China is on the way. Its achievements can, at least to a certain extent, switch the DUSEL from traditional people’s impression (exploitation and extraction) into a new care (conservation) to our Mother Earth.
